# Influence of Potassium Ions on Act of Amphotericin B to the DPPC/Chol Mixed Monolayer at Different Surface Pressures

**DOI:** 10.3390/membranes12010084

**Published:** 2022-01-13

**Authors:** Juan Wang

**Affiliations:** 1 School of Physics and Information Technology, Shaanxi Normal University, Xi’an 710100, China; wjbiophysics@yeah.net; 2 Shaanxi Engineering Research Center of Controllable Neutron Source, School of Science, Xijing University, Xi’an 710123, China

**Keywords:** amphotericin B, Langmuir monolayer, Langmuir–Blodgett film, potassium ions

## Abstract

Amphotericin B (AmB) is an antifungal drug that rarely develops resistance. It has an affinity with the cholesterol on mammalian cell membranes, disrupting the structure and function of the membranes, which are also affected by potassium ions. However, the mechanism is unclear. In this paper, the Langmuir monolayer method was used to study the effects of potassium ions on the surface pressure–mean molecular area of isotherms, elastic modulus and the surface pressure–time curves of a 1,2-dipalmitoyl-sn-glycero-3-phosphocholine/cholesterol (DPPC/Chol) monolayer and a DPPC/Chol/AmB monolayer. The morphology and thickness of the Langmuir–Blodgett films were studied via atomic force microscopy. The results showed that AmB can increase the mean molecular area of the DPPC/Chol mixed monolayer at low pressures (15 mN/m) but reduces it at high pressures (30 mN/m). The potassium ions may interfere with the effect of AmB in different ways. The potassium ions can enhance the influence of AmB on the stability of monolayer at low surface pressures, but weaken it at high surface pressures. The potassium ions showed significant interference with the interaction between AmB and the cholesterol-enriched region. The results are helpful for us to understand how the effect of amphotericin B on the phospholipid membrane is interfered with by potassium ions when amphotericin B enters mammalian cell membrane.

## 1. Introduction

More and more patients have undergone organ or bone marrow transplantation, which is accompanied by impaired immune function. There are also malignant diseases, AIDS and a variety of congenital or acquired autoimmune diseases, which create a risk of immune deficiency. Patients are at significant risk of systemic fungal infection, which can be life threatening [1]. However, the treatment of fungal infections presents considerable challenges because of the limited number of antifungal agents available [2] and because clinicians face many limitations related to the use of antifungal agents [3]. Amphotericin B (AmB) has been of great concern. Its most important advantage is that amphotericin B rarely develops resistance to fungi [4,5], but it is prone to severe nephrotoxicity in patients [6,7]. This is related to its pore-forming activity on membranes. Meanwhile, studies have shown that the polyene compound AmB induces changes in the permeability of mammalian immune cell membranes (forming membrane pores), leading to the increased expressions of pro-inflammatory cytokines and chemokines [8,9]. This means that AmB can be used as a potential new immunotherapy anticancer drug [10,11]. How amphotericin B affects cell membrane permeability is closely related to its pore-forming activity, but the mechanism is still unclear. Therefore, the modification of known antifungal agents and the development of new agents are of great value in the treatment of increasing numbers of systemic fungal infections. How to reduce the toxicity of polyene drugs to mammalian cells and improve their clinical efficacy has become an important medical issue. Studies of the pore-forming activity of amphotericin B on biofilms not only play an important role in the investigation of its antifungal activity and the mechanism of cell membrane toxicity, but also provide a fundamental guide for the development of amphotericin B as a new agent or subject of structural modification. Meanwhile, it has potential research value for the use of AmB in the immunotherapy of tumors in the future.

AmB consists of a heptacene macrolide skeleton, with a functional carboxyl group at position C16 and a mycosamine sugar appendage at position C19 [12]. The amphotericin B molecule has hydrophilic and hydrophobic properties. The hydrophilic region consists of one side of a ring (carbons 3–15) containing several hydroxyl groups. The other side of the ring (carbons 20–33) contains multiple double bonds and is hydrophobic [13]. The special effect of AmB on fungi is due to its greater affinity for ergosterol than cholesterol [14]. However, amphotericin B interacts with the cholesterol on the human cell membrane, affecting the permeability of the cell membrane and having a toxic effect on the cell membrane [15,16]. The mechanism of the toxicity of amphotericin B to human cell membranes is not clear. The interaction of amphotericin B with cholesterol-rich cell membranes is worthy of further investigation.

The toxicity of AmB to cholesterol-containing membranes has been studied extensively by many scholars. It has been found that AmB can combine cholesterol to form the complexes in lipid membranes [17,18,19], producing some highly structured pores [20]. AmB can initiate a redistribution of the cholesterol molecules in the plane of the membrane [21]. The penetration ability of AmB with the 1,2-dipalmitoyl-sn-glycero-3-phosphocholine (DPPC)/cholesterol (Chol) monolayer depends on the ratio of the two membrane components in the mixed monolayer and the strength of the interaction between the DPPC and Chol molecules [22]. Amphotericin B can cause an increase in the thickness of POPC (a kind of phospholipids, 1-palmitoyl-2-oleoyl-sn-glycero-3-phosphocholine) and POPC-sterol membranes [23]. Cholesterol markedly inhibits the ion permeability induced by AmB, which is because the thickness of the membrane affects the action of amphotericin B [24]. Interestingly, a metal cation may also affect AmB molecules’ aggregation on the membrane [25], particularly for potassium ions. M. Gagoś et al. suggested that the K^+^ ions can affect the aggregation levels of AmB molecules [26,27] studied using the different spectroscopic techniques, such as electronic absorption spectroscopy, Raman and FTIR. Additionally, the presence of K^+^ ions can facilitate the interaction between AmB molecules and lipid membranes [28]. J. Brajtburg et al. found that the ATP (Na^+^–K^+^) activity in animal cells may be inhibited by AmB [29] and that the K^+^ ions exhibited protective properties against the antifungal activity of AmB [30]. Therefore, it is important to understand the membrane toxicity of amphotericin B by investigating the effect of potassium ions on the membrane interaction.

The study of the interaction between drugs and model membranes can be used as a complementary method for the study of pharmacodynamics or toxicology of cell membranes in vitro and in vivo [31,32]. The Langmuir monolayer is currently a popular interfacial biomembrane model for providing an understanding of drug–biomembrane interactions on a molecular level [33,34,35]. Although the monolayer has only one lipid leaflet of the cell membrane, the simulation experiment using the Langmuir technology has the great advantage of being able to adjust the fluidity of a membrane, the surface pressure and the environment that the cell membrane is in [36]. In addition, the Langmuir–Blodgett method [37] can be used to transfer the interfacial monolayers onto the substrate to form the Langmuir–Blodgett films (LB films), and the films can be observed using atomic force microscopy [38] or scanning electron microscopy [39], which can provide some morphological information. Therefore, studying the interaction between the drug and the interfacial lipid monolayer model can help to understand the toxicity mechanism of amphotericin B as it affects the cell membrane on a molecular level.

When amphotericin B penetrates the cell membrane, it first interacts with the outer layer of the cell membrane. Phosphatidylcholine (PC) is the main phospholipid of the outer cell membrane leaflet [40] and the major component of lung surfactants in the human body [41]. Moreover, 1,2-dipalmitoyl-sn-glycero-3-phosphocholine (DPPC) is a saturated phospholipid and comprises about 30–60% of the PC [42]. Cholesterol is the main sterol of a mammalian plasma membrane, and can regulate the fluidity of the membrane [43]. More importantly, cholesterol is related to the toxicity of amphotericin B to the cell membrane. In this work, the DPPC/Chol (molar ratio, 7:3) mixed monolayer was selected to be a model of the outer the cell membrane leaflet, and the ratio was close to the real lipid/sterol ratio of a mammalian plasma membrane [44]. The molar fraction of AmB on the mixed monolayer was 50%, in which condition the attractive force of the molecules on the monolayer is the strongest according to our previous research [45]. The surface pressure–mean molecular area isotherms, elastic modulus and the stability of the DPPC/Chol mixed monolayer and the DPPC/Chol/AmB mixed monolayer were studied using the Langmuir method in the absence and presence of K^+^ ions. Additionally, the morphology and the thickness of the mixed Langmuir–Blodgett films were analyzed using atomic force microscopy.

## 2. Materials and Methods

### 2.1. Materials

First, 1,2-dipalmitoyl-sn-glycero-3-phosphocholine (DPPC: purity ≥ 99%), cholesterol (Chol: purity ≥ 99%) and power amphotericin B (AmB: purity > 80%) were purchased from Sigma, USA. The mica sheets were purchased from Xi’an Qiyue Biotechnology Company, China. The other chemicals were of analytical grade and were used without further purification. The DPPC and Chol (7:3, mol/mol) were dissolved in a chloroform/methanol mixture (9:1, *v*/*v*) to obtain a lipid monolayer-forming solution, and its final concentration was 0.5 mmol/L. The AmB molecules were dissolved in a 3:1 (*v*/*v*) mixture of dimethylformamide and 1 M HCl to give a final concentration of 0.5 mmol/L. High purity water obtained from a Milli-Q plus water purification system (18.2 MΩ/cm, Millipore, MA, USA) was used in all experiments. Potassium carbonate was dissolved in HEPES buffer (pH 7) to form subphase solutions with different concentrations (0 mmol/L, 5 mmol/L and 10 mmol/L) of potassium ions.

### 2.2. Langmuir Monolayers

The Langmuir monolayer experiments were performed using a Langmuir trough (Teflon trough size 323 mm × 75 mm × 5 mm, KSV-Minitrough, Helsinki, Finland). The surface pressure–mean molecular area (π-A) isotherms of the DPPC/Chol mixed monolayer and the DPPC/Chol/AmB mixed monolayer were measured with a Wilhelmy-type tensiometer using filter paper (10 mm × 30 mm × 0.15 mm) attached to the trough. The accuracy of the sensor was 0.1 mN/m. Before each experiment, we washed the trough with ethanol and rinsed it with purified water. To confirm that the surface of the trough and subphase were adequately cleaned, we compressed the barriers over the entire surface area range to ensure that surface pressure fluctuations were less than ±0.2 mN/m [46]. For all the experiments, the temperature was maintained at 37 ± 1 °C by an external circulator, and the trough was filled with normal saline with a 0 mmol/L, 5 mmol/L or 10 mmol/L potassium ions solution as the subphase. For the DPPC/Chol mixed monolayer, 50 μL mixed lipid solution was deposited at the air–water interface with a Hamilton micro-syringe. For the DPPC/Chol/AmB mixed monolayer (lipid/AmB in 1:1 molar radio), 25 μL lipid solution and 25 μL AmB solution were spread on the interface. We waited 15 min to make sure that all the solvent evaporated, and compressed the monolayers with a barrier speed of 75 cm^2^/min. The surface pressure was recorded during the interface compression process. The total number of moles remained identical in both monolayer systems.

The surface pressure–time (π-t) curves of the DPPC/Chol mixed monolayer and the DPPC/Chol/AmB mixed monolayer were obtained as follows. The lipid molecules or and AmB molecules were dispersed on the interface. After 15 min, we compressed the monolayer to make the surface pressure reach a certain surface pressure (15 or 30 mN/m), and then kept the area of the monolayer constant [47]. The surface pressure–time (π-t) curve of the lipid monolayer or the lipid–AmB monolayer was recorded.

All of the π-A isotherms and π-t curves were repeated three times independently to confirm the reproducibility of the measurements, and the final data were averaged.

### 2.3. Langmuir-Blodgett Film and Atomic Force Microscopy Observation

The Langmuir monolayer at the air–water interface can be transferred onto the substrate to form the Langmuir–Blodgett film. At first, the freshly cleaved mica sheet was half-dipped into the subphase vertically. Then, we spread the lipid molecules or the AmB molecules on the interface and compressed the monolayer after 15 min for solvent evaporation. When the surface pressure reached up to 15 or 30 mN/m, we pulled the mica sheet out from the subphase vertically at the dipping rate of 5 mm/min. In this process, the monolayer was deposited onto the surface of the mica sheet to form a Langmuir–Blodgett film. The films relative to the subphase with 0 mmol/L, 5 mmol/L and 10 mmol/L potassium ions were studied.

The microstructure characterization of the Langmuir–Blodgett film was observed via the atomic force microscopy (AFM, Shimadzu, Tokyo, Japan) in the intermittent contact mode using a silicon nitride pyramidal tip mounted on a 100 μm long cantilever with a force constant of 0.1 N/m.

## 3. Results

### 3.1. The Surface Pressure–Mean Molecular Area (π-A) Isotherms and the Elastic Modulus $C_{s}^{-1}$

**Figure 1:**
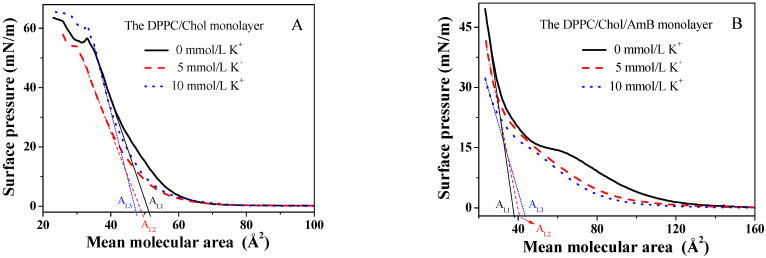
The surface pressure–mean molecular area (π-A) isotherms of the (**A**). DPPC/Chol mixed monolayer and (**B**). the DPPC/Chol/AmB mixed monolayer at the air–water interface in the absence and presence of potassium ions (5 mmol/L and 10 mmol/L).

The surface pressure–mean molecular area isotherms of the DPPC/Chol mixed monolayer rose up when the mean molecular area was about 65 ± 0.01$\;{Å}^{2}$, which is similar to the literature [48,49], and which was independent of the presence or absence of potassium ions (Figure 1A). The isotherms in the absence of the potassium ions dropped sharply at the surface pressure of 56.6 mN/m, corresponding to the collapse of the DPPC/Chol mixed monolayer, which meant that the monolayer may have broken down. At the surface pressure higher than 56.6 mN/m, the isotherms rose again, meaning that the monolayer may have formed a double-layered structure due to the extrusion. The collapse pressure decreased in the presence of 5 mmol/L K^+^ ions, but increased in the presence of 10 mmol/L K^+^ ions. In the isotherms of the DPPC/Chol mixed monolayer, an obvious phase transfer process was not observed, which was similar to the results in the literature [45]. This was not affected by potassium ions.

**Figure 2:**
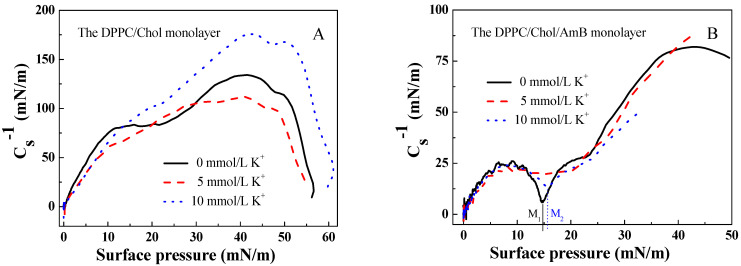
The elastic modulus–surface pressure ($C_{s}^{-1}$-π) curves of (**A**). the DPPC/Chol mixed monolayer and (**B**). the DPPC/Chol/AmB mixed monolayer at the air–water interface in the absence and presence of potassium ions (5 mmol/L and 10 mmol/L).

Compared to that in the absence of the K^+^ ions, the π-A isotherms of the DPPC/Chol/AmB mixed monolayer in the presence of K^+^ ions had a lower lift-off area (100$\;\pm \;0.01\;{Å}^{2}$ as compared to 130$\;\pm \;0.01\;{Å}^{2}$), as shown in Figure 1B. The lower lift-off areas show that the mean molecular area of the mixed monolayer was smaller in the LE phase caused by the K^+^ ions. It is worth noting that in the absence of the K^+^ ions, the isotherm had a flat region on the isotherm of the DPPC/Chol/AmB mixed monolayer. In our previous study [50], a flat platform was also observed on the pure AmB monolayer, which corresponded to the phase transition from the G (gas)–LE (liquid expanded) coexistence phase to the LE phase. That was due to the orientation of the AmB molecule at the interface. Thus, in Figure 1B, the presence of the flat platform was caused by the rearrangement of the AmB molecules in the lipid monolayer. In the presence of 5 mmol/L K^+^ ions, the flat platform disappeared. However, in the presence of 10 mmol/L K^+^ ions, the flat platform was not obvious, but the phase transition corresponding to the flat platform was observed on the $C_{s}^{-1}$-π curves (Figure 2B). This suggested that the K^+^ ions may directly induce the change in AmB’s molecular conformation.

According to the data of the π-A isotherms of the DPPC/Chol mixed monolayer with and without AmB molecules, the elastic modulus of the monolayer can be calculated using the formula [51,52]:

(1) $$C_{s}^{-1}=-A{\left(\frac{d\pi }{dA}\right)}_{T}$$

where $C_{s}^{-1}$ is the elastic modulus of the monolayer, *s* is the cross-sectional area of the monolayer, *A* is the mean molecular area and *π* is the surface pressure of the monolayer. A greater elastic modulus suggests that the monolayer is less compressible and more condensed [53]. The minimum of $C_{s}^{-1}$ indicates a significant phase transition in the monolayer [54].

From Figure 2, the maximum value of $C_{s}^{-1}$ for the DPPC/Chol mixed monolayer was greater than that for the DPPC/Chol/AmB mixed monolayer, and it did not matter if potassium ions were present or not. It was suggested that AmB caused the DPPC/Chol mixed monolayer to be less condensed. In Figure 2A, the maximum value of $C_{s}^{-1}$ for the DPPC/Chol mixed monolayer decreased due to the presence of 5 mmol/L K^+^ ions, but increased because of the presence of 10 mmol/L K^+^ ions. The potassium ions can affect the molecular arrangement on the DPPC/Chol mixed monolayer, which was different depending on its concentration. The potassium caused the DPPC/Chol mixed monolayer to become more condensed in the concentration of 10 mmol/L, but induced the mixed monolayer to become less condensed in the concentration of 5 mmol/L. However, for the DPPC/Chol/AmB mixed monolayer, the potassium ions caused the monolayer to become more condensed in the concentration of 5 mmol/L, but induced the mixed monolayer to become less condensed in the concentration of 10 mmol/L.

There was no minimum of $C_{s}^{-1}$ appearing on the $C_{s}^{-1}$-π curves of the DPPC/Chol mixed monolayer in the absence and presence of potassium ions. The $C_{s}^{-1}$ value of the DPPC/Chol/AmB mixed monolayer reached a minimum at the surface pressure of 14.8 mN/m ($M_{1}$ in Figure 2B) in the absence of potassium ions and at the surface pressure of 15.7 mN/m ($M_{2}$ in Figure 2B) in the presence of 10 mmol/L K^+^ ions. However, the potassium ions in the concentration of 5 mmol/L caused the minimum of $C_{s}^{-1}$ to disappear.

### 3.2. The Limiting Molecular Area and the Mean Molecular Area Increment

According to the π-A isotherms, the limiting molecular area can be calculated just by extrapolating the linear part of the curve according to the lowest compressibility, which is taken as the region of the surface pressure corresponding to the linear part [55]. The limiting molecular area of the mixed monolayer in the absence of the K^+^ ions and in the presence of 5 mmol/L and 10 mmol/L K^+^ ions is marked as $A_{L1}$, $A_{L2}$ and $A_{L3}$ in Figure 1. The K^+^ ions decreased the limiting molecular area of the DPPC/Chol mixed monolayer, and the limiting molecular area was smallest in the presence of 10 mmol/L K^+^ ions. However, the K^+^ ions increased the limiting molecular area of the DPPC/Chol/AmB mixed monolayer, and the limiting molecular area was the largest in the presence of 10 mmol/L K^+^ ions. This indicated that the potassium ions may affect the interaction between AmB and the lipid molecules, and that the AmB molecules were sensitive to potassium ions.

**Figure 3:**
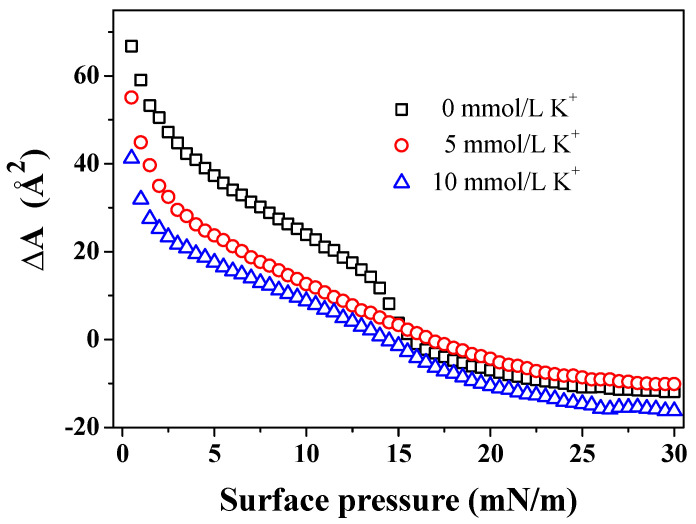
The mean molecular area increment (∆A) of the DPPC/Chol mixed monolayer caused by AmB in the absence and presence of potassium ions at the same surface pressure.

The AmB molecules can influence the mean molecular area of the DPPC/Chol mixed monolayer, as shown in Figure 3. According to the data of π-A isotherm, the mean molecular area increment (∆A) value is $A_{DPPC/Chol/AmB}-A_{DPPC/Chol}$. The positive value of ∆A suggests that AmB can increase the mean molecular area of the DPPC/Chol mixed monolayer, and the negative value of ∆A indicates that AmB can decrease it. In the absence of K^+^ ions, AmB increased the mean molecular area of the DPPC/Chol mixed monolayer in the range of the surface pressure from 0 mN/m to 15.5 mN/m, but decreased it in the range of the surface pressure from 15.5 mN/m to 30 mN/m, which was similar than that in the presence of K^+^ ions. However, the ranges of the surface pressure were slightly different. In the presence of 5 mmol/L K^+^ ions, the AmB molecules increased the mean molecular area of the lipid mixed monolayer at lower levels of surface pressure of 17 mN/m (14.5 mN/m in the presence of 10 mmol/L K^+^ ions) but decreased it at the surface pressures from 17 mN/m to 30 mN/m (from 14.5 mN/m to 30 mN/m in the presence of 10 mmol/L K^+^ ions). Remarkably, at lower levels of surface pressure, the ∆A values in the presence of K^+^ ions were smaller than that in the absence of K^+^ ions, and the ∆A values caused by 10 mmol/L K^+^ ions were the smallest at the same surface pressure. It was suggested that the effect of AmB increasing the mean molecular area in the presence of 10 mmol/L K^+^ ions was weakest. At the higher levels of surface pressure, the |∆A| value was largest in the presence of 10 mmol/L K^+^ ions and it was slightly smaller in the presence of 5 mmol/L K^+^ ions than that in the absence of the K^+^ ions. It was suggested that 5 mmol/L K^+^ ions weakened the AmB’s ability to decrease the mean molecular area, but the 10 mmol/L K^+^ ions enhanced the AmB’s ability at higher levels of surface pressure.

### 3.3. The Surface Pressure–Time (π-t) Curves

**Figure 4:**
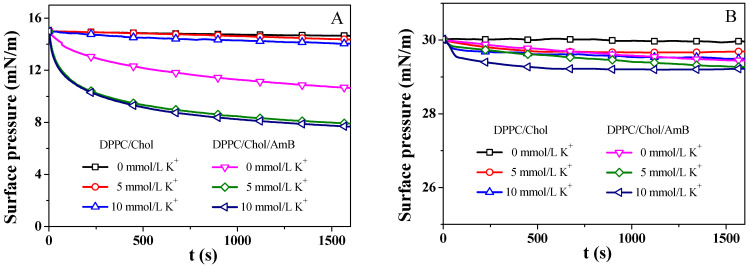
The surface pressure–time (π-t) curves of the DPPC/Chol mixed monolayer and the DPPC/Chol/AmB mixed monolayer at the air–water interface in the absence and presence of potassium ions (5 mmol/L and 10 mmol/L) at 15 mN/m (**A**) and 30 mN/m (**B**).

The DPPC/Chol mixed monolayer and the DPPC/Chol/AmB mixed monolayer were compressed to a surface pressure of 15 mN/m and 30 mN/m, and the area of the monolayer was kept constant. We recorded the change in the surface pressure over time, as seen in Figure 4. The surface pressure was decreased or increased until the equilibrium value ($\pi_{e}$) was reached [56]. Then, $∆\pi_{e}$ was calculated using the formula: $∆\pi_{e}=\pi_{e-AmB/lipid}-\pi_{e-lipid}$, which indicated the change in $\pi_{e}$ (Table 1). A positive $∆\pi_{e}$ value indicates that the stability of the monolayer was enhanced by AmB, while a negative $∆\pi_{e}$ value suggests that the stability of the monolayer was weakened by AmB [56]. A smaller value of $\left|∆\pi_{e}\right|$ means a weaker impact of AmB on the stability of the lipid monolayer.

At 15 mN/m, the potassium ions decreased the $\pi_{e}$ value of the DPPC/Chol mixed monolayer, suggesting that K^+^ ions can decrease the stability of the DPPC/Chol mixed monolayer, which did not depend on the presence of AmB molecules. The AmB molecules significantly decreased the $\pi_{e}$ value of the DPPC/Chol mixed monolayer in the absence of potassium ions, indicating that the stability of the DPPC/Chol mixed monolayer was decreased by the AmB drug, and that the presence of potassium ions can enhance this effect of AmB. At 30 mN/m, the presence of AmB and potassium ions slightly reduced the stability of the monolayers, but the potassium ions slightly weakened the impact of AmB on the stability of the lipid monolayer.

### 3.4. The AFM Analysis

**Figure 5:**
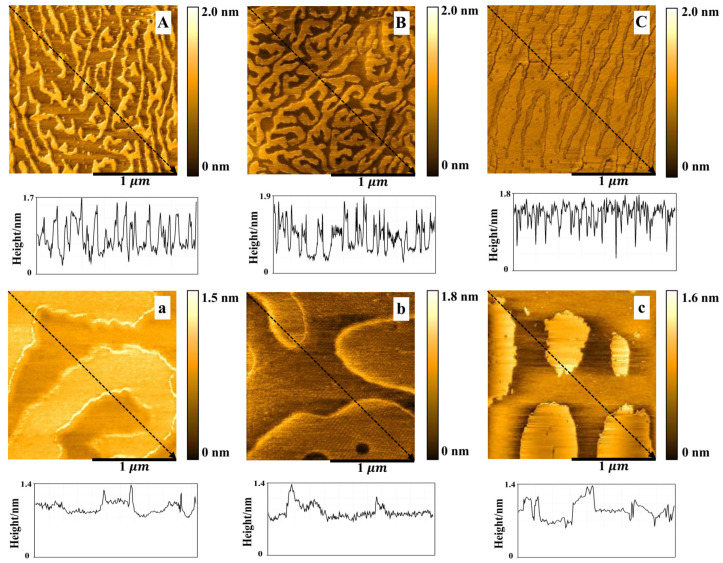
The AFM images (2 μm×2 μm) of the DPPC/Chol mixed Langmuir–Blodgett film without (**A**–**C**) and with AmB (**a**–**c**) in the absence (**A**,**a**) and presence of 5 mmol/L (**B**,**b**) and 10 mmol/L (**C**,**c**) K^+^ ions at 15 mN/m.

The DPPC/Chol mixed monolayers were transferred onto the surface of mica sheets at the surface pressure of 15 mN/m. The bright (higher) areas and the dark (lower) areas were observed on the DPPC/Chol film (Figure 5A–C), which was highly similar to the results of A. Botet-Carreras et al. [57]. The bright areas may be attributed to the segregation of enriched Chol domains [57]. In the absence of K^+^ ions, the enriched Chol domains in the DPPC/Chol mixed monolayer film formed a closely arranged “curved dendritic” shape, the height of which was about 1.3~1.7 nm (Figure 5A). In the presence of 5 mmol/L K^+^ ions, the morphology did not change, but the enriched Chol regions were more compact than that in the absence of the K^+^ ions, and the height was about 1.1~1.9 nm (Figure 5B). In the presence of 10 mmol/L K^+^ ions, the morphology of the DPPC/Chol mixed monolayer film in the Chol-rich regions changed to an “uncurved dendritic” shape with the height of about 1.1~1.8 nm (Figure 5C). The dark region was at the height of 0.3~0.6 nm in the absence of K^+^ ions. In the presence of AmB, the bright area of the lipid monolayer was shaped similarly to a large island with a height of about 1.0~1.4 nm (Figure 5a–c). The height of the dark region was 0.6~0.8 nm. As the potassium ions’ concentration increased, the area of the lower region seemed to increase slightly.

AmB increased the height of the dark region of the DPPC/Chol mixed LB film at 15 mN/m, which was not affected by potassium ions. However, AmB reduced the height of the bright region of the DPPC/Chol mixed LB film, which was interfered with by potassium ions. In the absence of potassium ions, the height of the bright area decreased by 0.3 nm, but in the presence of 5 mmol/L and 10 mmol/L potassium ions, the height decreased by 0.1–0.5 nm and 0.1–0.4 nm, respectively. It can be seen that potassium ions can interfere with the interaction between AmB and the cholesterol-enriched region at 15 mN/m.

**Figure 6:**
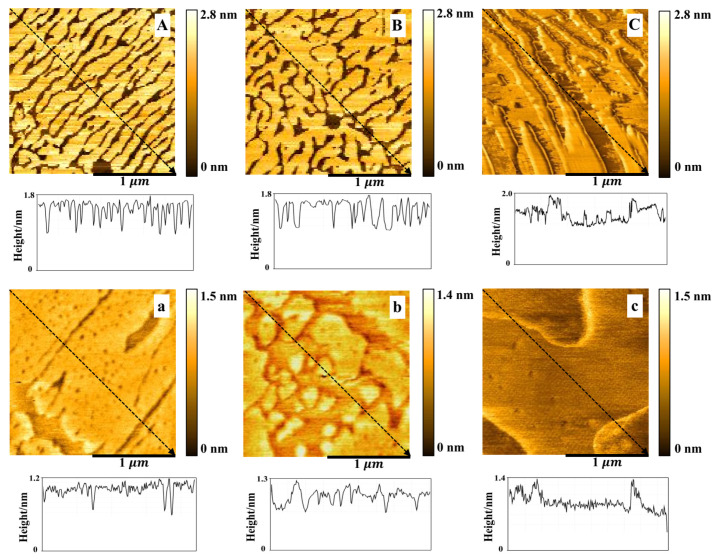
The AFM images (2 μm×2 μm) of the DPPC/Chol mixed Langmuir–Blodgett film without (**A**–**C**) and with AmB (**a**–**c**) in the absence (**A**,**a**) and presence of 5 mmol/L (**B**,**b**) and 10 mmol/L (**C**,**c**) K^+^ ions at 30 mN/m.

From the Figure 6, the DPPC/Chol mixed monolayers, with and without AmB, were more compact at 30 mN/m than that at 15 mN/m. The height of the bright region (enriched Chol) of the DPPC/Chol mixed monolayer was about 1.6~1.8 nm and the height of dark region was about 0.9 nm in the absence and presence of 5 mmol/L K^+^ ions. In the presence of 10 mmol/L K^+^ ions, the bright region was at the height of about 1.5~2.0 nm and the dark region was at the height of about 1.0 nm. For the DPPC/Chol/AmB mixed monolayer, the height of the bright region was 1.0~1.2 nm in the absence of the K^+^ ions, 1.0~1.3 nm in the presence of 5 mmol/L K^+^ ions and 1.0~1.4 nm in the presence of 10 mmol/L K^+^ ions. The dark region was at the height of about 0.6~0.8 nm in the absence of K^+^ ions and about 0.7~0.9 nm in the presence of K^+^ ions.

AmB reduced the height of both bright and dark regions of the DPPC/Chol mixed LB film at 30 mN/m, but the effect of AmB on the height of the bright regions was slightly disturbed by the potassium ions. In the absence of potassium ions, AmB reduced the height of the bright regions by 0.4–0.6 nm, but in the presence of potassium ions, the height decreased by 0.5–0.6 nm. It indicated that potassium ions had a slight effect on the interaction between AmB and the cholesterol-enriched region.

**Figure 7:**
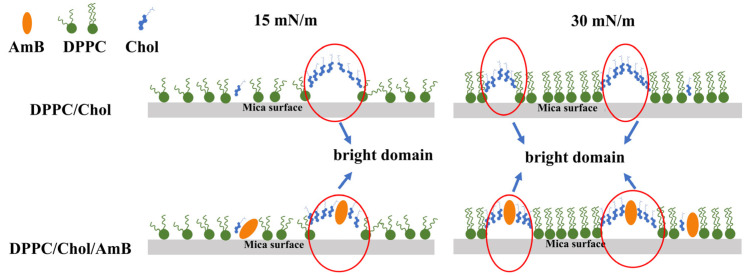
Illustration representing the possible organization of the DPPC/Chol mixed LB film and the DPPC/Chol/AmB mixed LB film.

An illustration of the possible molecular arrangement of the DPPC/Chol mixed LB film and the DPPC/Chol/AmB mixed LB film is shown in Figure 7. At 15 mN/m, the bright domains of the DPPC/Chol mixed LB film may be attributed to the segregation of enriched cholesterol domains. At the dark domains, cholesterol did not accumulate. The surface pressure of 15 mN/m corresponded to the phase transition of the AmB component, so the orientation of the AmB molecules on the interface may change from horizontal to vertical, and the longitudinal axis of most AmB molecules may have a certain inclination angle with the interface. For the DPPC/Chol/AmB mixed LB film, AmB mainly expressed an affinity for cholesterol molecules. At the lower domain, the cholesterol molecules did not aggregate, and the longitudinal axis of the AmB molecules was obliquely inclined to the interface. At the higher domain, cholesterol aggregated, and the vertical axis of the AmB molecule was vertical. At 30 mN/m, the molecules were packed together, the cholesterol molecules were more concentrated, and the orientation of the AmB was vertical. Therefore, according to the height analysis of the AFM images, the potassium ions may interfere with the interaction between AmB and the cholesterol-rich region, which was stronger at 15 mN/m than that at 30 mN/m. Moreover, the slight disturbance caused by the potassium ions was almost not effected by the concentration of the potassium ions at 30 mN/m.

## 4. Discussion

According to the results, AmB can increase the mean molecular area of DPPC/Chol mixed monolayer at low pressures (15 mN/m). The 5 mmol/L K^+^ ions had little effect on AmB’s activities, but 10 mmol/L K^+^ ions can weaken the effect of AmB. In contrast, AmB can reduce the mean molecular area of DPPC/Chol mixed monolayers at high pressures (30 mN/m). The 5 mmol/L K^+^ ions slightly attenuated AmB’s effects, but the 10 mmol/L K^+^ ions enhanced it. Regardless of the surface pressure, AmB can reduce the stability of monolayers. However, potassium ions interfered with AmB in different ways at different surface pressures. At low surface pressures, potassium ions can significantly enhance the influence of AmB on the stability of monolayers, and the interference of potassium was stronger at the concentration of 5 mmol/L. At high surface pressures, potassium ions weaken the effect of AmB on the stability of monolayers. At the same time, the higher the concentration of potassium ions, the more its interference was obvious. On the DPPC/Chol LB film, there were cholesterol-rich regions. When AmB interacted with the lipid components, a large number of AmB molecules interacted with the cholesterol-enriched region due to the strong affinity of AmB molecules for cholesterol molecules. The potassium ions showed significant interference of the interaction between AmB and cholesterol-enriched region, which led to the change in the film’s thickness. Additionally, when the surface pressure was lower, the interference effect was stronger. However, the potassium ions had little effect on the interaction between AmB and the region with low cholesterol on the film.

## 5. Conclusions

The interaction between amphotericin B and cell membranes is complex. In this paper, a simplified phospholipid membrane model was used to study the effect of amphotericin B on the physical properties and membrane structure of cell membranes in a potassium ion environment. When amphotericin B interacts with the cell membrane, it enters the outer layer first. Amphotericin B can change the elastic modulus, morphology and thickness of the outer layer of a membrane, which is, itself, affected by the concentration of potassium ions in the environment. At the same time, when the membrane performs its normal physiological function, the curvature of the membrane may change, along with the surface pressure of the membrane. The potassium ions can interfere with the effect of amphotericin B on the stability of the membrane, as well as with its effects on the morphology and thickness of the membrane, which provides more favorable information for understanding the toxicity of AmB to the membrane of cells. Next, we will focus on the potential stability of the AmB interacting with lipid monolayer systems. In addition, the species and concentration of ions in the environment of the membrane should be emphasized in the study of drug–membrane interaction. Moreover, the two-layer membrane model of the vesicle can be used, which has an interface curvature similar to that of the cell membrane, and different ion concentrations can be introduced inside and outside the vesicle membrane. One must make it as close to the real membrane environment as possible.

## Figures and Tables

**Table 1:** The equilibrium values ($\pi_{e}$) of the surface pressure for the DPPC/Chol mixed monolayer ($\pi_{e-lipid}$ ) and the DPPC/Chol/AmB monolayer ($\pi_{e-AmB/lipid}$ ) at 15 mN/m and 30 mN/m. The change of $\pi_{e}$ is $∆\pi_{e}\left(\pi_{e-AmB/lipid}-\pi_{e-lipid}\right)$.

Concentration of K^+^ (mmol/L)	15 mN/m			30 mN/m		
	$\pi_{e-lipid}\;(\mathrm{mN}/m)$	$\pi_{e-AmB/lipid}\;(\mathrm{mN}/m)$	$∆\pi_{e}\;(\mathrm{mN}/m)$	$\pi_{e-lipid}\;(\mathrm{mN}/m)$	$\pi_{e-AmB/lipid}\;(\mathrm{mN}/m)$	$∆\pi_{e}\;(\mathrm{mN}/m)$
0	14.62 ± 0.02	9.83 ± 0.02	−4.79 ± 0.02	29.92 ± 0.02	29.45 ± 0.02	−0.47 ± 0.02
5	13.88 ± 0.02	7.11 ± 0.02	−6.77 ± 0.02	29.69 ± 0.02	29.27 ± 0.02	−0.42 ± 0.02
10	13.53 ± 0.02	6.91 ± 0.02	−6.62 ± 0.02	29.49 ± 0.02	29.21 ± 0.02	−0.28 ± 0.02

## References

[1] Reinhart K., Bauer M., Riedemann N.C., Hartog C.S. (2012). New Approaches to Sepsis: Molecular Diagnostics and Biomarkers. Clin. Microbiol. Rev..

[2] Seyedmousavi S., Rafati H., Ilkit M., Tolooe A., Hedayati M.T., Verweij P. (2016). Systemic Antifungal Agents: Current Status and Projected Future Developments. Methods Mol. Biol..

[3] Wang J.-L., Chang C.-H., Young-Xu Y., Chan K. (2010). Systematic Review and Meta-Analysis of the Tolerability and Hepatotoxicity of Antifungals in Empirical and Definitive Therapy for Invasive Fungal Infection. Antimicrob. Agents Chemother..

[4] Pfaller M.A., Espinel-Ingroff A., Canton E., Castanheira M., Cuenca-Estrella M., Diekema D., Fothergill A., Fuller J., Ghannoum M., Jones R.N. (2012). Wild-Type MIC Distributions and Epidemiological Cutoff Values for Amphotericin B, Flucytosine, and Itraconazole and Candida spp. as Determined by CLSI Broth Microdilution. J. Clin. Microbiol..

[5] Fanos V., Cataldi L. (2001). Renal Transport of Antibiotics and Nephrotoxicity: A Review. J. Chemother..

[6] Wong-Beringer A., Jacobs R.A., Guglielmo B.J. (1998). Lipid Formulations of Amphotericin B: Clinical Efficacy and Toxicities. Clin. Infect. Dis..

[7] Laniado-Laborín R., Cabrales-Vargas M.N. (2009). Amphotericin B: Side effects and toxicity. Rev. Iberoam. Micol..

[8] Baek S., Kim S.-M., Lee S.-A., Rhim B.-Y., Eo S.-K., Kim K. (2013). The Cholesterol-Binding Antibiotic Nystatin Induces Expression of Macrophage Inflammatory Protein-1 in Macrophages. Biomol. Ther..

[9] Rogers P.D., Pearson M.M., Cleary J.D., Sullivan D.C., Chapman S.W. (2002). Differential expression of genes encoding immunomodulatory proteins in response to amphotericin B in human mononuclear cells identified by cDNA microarray analysis. J. Antimicrob. Chemother..

[10] Kim N., Choi J.-W., Park H.-R., Kim I., Kim H.S. (2017). Amphotericin B, an Anti-Fungal Medication, Directly Increases the Cytotoxicity of NK Cells. Int. J. Mol. Sci..

[11] Zhang J., Cao D., Yu S., Chen L., Wei D., Shen C., Zhuang L., Wang Q., Xu X., Tong Y. (2018). Amphotericin B suppresses M2 phenotypes and B7-H1 expression in macrophages to prevent Raji cell proliferation. BMC Cancer.

[12] Ermishkin L.N., Kasumov K.M., Potzeluyev V.M. (1976). Single ionic channels induced in lipid bilayers by polyene antibiotics amphotericin B and nystatine. Nature.

[13] García-Barbazán I., Zaragoza Ó. (2021). Polyenes and Amphotericin B. Encycl. Mycol..

[14] Kotler-Brajtburg J., Price H.D., Medoff G., Schlessinger D., Kobayashi G.S. (1974). Molecular Basis for the Selective Toxicity of Amphotericin B for Yeast and Filipin for Animal Cells. Antimicrob. Agents Chemother..

[15] Thanki K., Prajapati R., Sangamwar A.T., Jain S. (2018). Long chain fatty acid conjugation remarkably decreases the aggregation induced toxicity of Amphotericin B. Int. J. Pharm..

[16] Starzyk J., Gruszecki M., Tutaj K., Luchowski R., Szlazak R., Wasko P., Grudzinski W., Czub J., Gruszecki W.I. (2014). Self-Association of Amphotericin B: Spontaneous Formation of Molecular Structures Responsible for the Toxic Side Effects of the Antibiotic. J. Phys. Chem. B.

[17] Silberstein A. (1998). Conformational Analysis of Amphotericin B—Cholesterol Channel Complex. J. Membr. Biol..

[18] Saka Y., Mita T. (1998). Interaction of Amphotericin B with Cholesterol in Monolayers, Aqueous Solutions, and Phospholipid Bilayers. J. Biochem..

[19] Seoane R., Minones J., Conde O., Iribarnegaray E., Casas M. (1999). Interactions between amphotericin B and sterols in monolayers. Mixed films of amphotericin B/cholesterol. Langmuir.

[20] De Kruijff B., Demel R. (1974). Polyene antibiotic-sterol interactions in membranes of Acholeplasma laidlawii cells and lecithin liposomes. III. Molecular structure of the polyene antibiotic-cholesterol complexes. Biochim. Biophys. Acta (BBA) Biomembr..

[21] Fournier I., Barwicz J., Auger M., Tancrède P. (2008). The chain conformational order of ergosterol- or cholesterol-containing DPPC bilayers as modulated by Amphotericin B: A FTIR study. Chem. Phys. Lipids.

[22] Dynarowicz-Łątka P., Seoane R., Miñones J., Velo M. (2003). Study of penetration of amphotericin B into cholesterol or ergosterol containing dipalmitoyl phosphatidylcholine Langmuir monolayers. Colloids Surf. B Biointerfaces.

[23] Foglia F., Drake A., Terry A., Rogers S., Lawrence M., Barlow D. (2011). Small-angle neutron scattering studies of the effects of amphotericin B on phospholipid and phospholipid–sterol membrane structure. Biochim. Biophys. Acta (BBA) Biomembr..

[24] Matsuoka S., Murata M. (2002). Cholesterol markedly reduces ion permeability induced by membrane-bound amphotericin B. Biochim. Biophys. Acta (BBA) Biomembr..

[25] Becucci L., Innocenti M., Bellandi S., Guidelli R. (2013). Permeabilization of mercury-supported biomimetic membranes by amphotericin B and the role of calcium ions. Electrochim. Acta.

[26] Gagoś M., Arczewska M. (2011). Influence of K+ and Na+ Ions on the Aggregation Processes of Antibiotic Amphotericin B: Electronic Absorption and FTIR Spectroscopic Studies. J. Phys. Chem. B.

[27] Gagoś M., Arczewska M., Gruszecki W.I. (2011). Raman Spectroscopic Study of Aggregation Process of Antibiotic Amphotericin B Induced by H+, Na+, and K+ Ions. J. Phys. Chem. B.

[28] Arczewska M., Gagos M. (2011). Molecular organization of antibiotic amphotericin B in dipalmitoylphosphatidylcholine monolayers induced by K^+^ and Na^+^ ions: The Langmuir technique study. Biochim. Et Biophys. Acta (BBA) -Biomembr..

[29] Brajtburg J., Bolard J. (1996). Carrier effects on biological activity of amphotericin B. Clin. Microbiol. Rev..

[30] Brajtburg J., Medoff G., Kobayashi G.S., Elberg S. (1980). Influence of extracellular K^+^ or Mg^2+^ on the stages of the antifungal effects of amphotericin B and filipin. Antimicrob. Agents Chemother..

[31] DeGorter M.K., Xia C.Q., Yang J.J., Kim R.B. (2012). Drug Transporters in Drug Efficacy and Toxicity. Annu. Rev. Pharmacol. Toxicol..

[32] Zhang G., Ma Y., Xi D., Rao Z., Sun X., Wu X. (2019). Effect of high uric acid on the disposition of metformin: In vivo and in vitro studies. Biopharm. Drug Dispos..

[33] Ferrreira J.V.N., Grecco S.D.S., Lago J.H.G., Caseli L. (2015). Ultrathin films of lipids to investigate the action of a flavonoid with cell membrane models. Mater. Sci. Eng. C.

[34] Pascholati C.P., Lopera E.P., Pavinatto F.J., Caseli L., Nobre T.M., Zaniquelli M.E., Viitala T., D’Silva C., Oliveira O.N. (2009). The interaction of an antiparasitic peptide active against African Sleeping Sickness with cell membrane models. Colloids Surf. B Biointerfaces.

[35] Goto T.E., Caseli L. (2014). The interaction of mefloquine hydrochloride with cell membrane models at the air–water interface is modulated by the monolayer lipid composition. J. Colloid Interface Sci..

[36] Brockman H. (1999). Lipid monolayers: Why use half a membrane to characterize protein-membrane interactions?. Curr. Opin. Struct. Biol..

[37] Nagamine M., Osial M., Widera-Kalinowska J., Jackowska K., Krysiński P. (2020). Photosensitive Thin Films Based on Drop Cast and Langmuir-Blodgett Hydrophilic and Hydrophobic CdS Nanoparticles. Nanomaterials.

[38] Nakahara H., Hagimori M., Mukai T., Shibata O. (2018). Monolayers of a tetrazine-containing gemini amphiphile: Interplays with biomembrane lipids. Colloids Surf. B Biointerfaces.

[39] Wang J., Sun R. (2016). Influence of alkaline phosphatase on phase state of the SM monolayers at the air-water interface. Colloids Surf. A: Physicochem. Eng. Asp..

[40] Wnętrzak A., Chachaj-Brekiesz A., Kuś K., Filiczkowska A., Lipiec E., Kobierski J., Petelska A.D., Dynarowicz-Latka P. (2021). 25-hydroxycholesterol interacts differently with lipids of the inner and outer membrane leaflet—The Langmuir monolayer study complemented with theoretical calculations. J. Steroid Biochem. Mol. Biol..

[41] González C.M., Pizarro-Guerra G., Droguett F., Sarabia M. (2015). Artificial biomembrane based on DPPC—Investigation into phase transition and thermal behavior through ellipsometric techniques. Biochim. Biophys. Acta (BBA) Biomembr..

[42] Zuo Y.Y., Veldhuizen R.A.W., Neumann A.W., Petersen N.O., Possmayer F. (2008). Current perspectives in pulmonary surfactant—Inhibition, enhancement and evaluation. Biochim. Biophys. Acta (BBA) Biomembr..

[43] Yang S.-T., Kreutzberger A., Lee J., Kiessling V., Tamm L.K. (2016). The role of cholesterol in membrane fusion. Chem. Phys. Lipids.

[44] Ruiz G.C.M., Pazin W.M., Morato L.F.D.C., Oliveira O.N., Constantino C.J.L. (2020). Correlating mono- and bilayers of lipids to investigate the pronounced effects of steroid hormone 17α-ethynylestradiol on membrane models of DPPC/cholesterol. J. Mol. Liq..

[45] Wang J., Sun R., Li J. (2016). Influence of K^+^, Na^+^ or Ca^2+^ ions on the interaction between AmB and saturated phospholipids by Langmuir technique. Chem. Res. Chin. Univ..

[46] Lalgee L.J., Cox L., Fairman R.A., Grierson L. (2018). DPPC monolayer response to non-spanning cobalt-cage metallosurfactants: Electrostatic complex formation. Chem. Phys. Lipids.

[47] Wang J., Shi R.X., Sun R.G., Hao C.C., Li J.H., Lu X.L. (2016). Influence of amphotericin B on liquid crystal state of the Cholesterol/Dipalmitoylphosphatidylcholine monolayer in the presence of different metal cations. Chin. Phys. B.

[48] Gong K., Feng S.-S., Go M.L., Soew P.H. (2002). Effects of pH on the stability and compressibility of DPPC/cholesterol monolayers at the air–water interface. Colloids Surf. A Physicochem. Eng. Asp..

[49] Guzmán E., Liggieri L., Santini E., Ferrari M., Ravera F. (2013). Mixed DPPC–cholesterol Langmuir monolayers in presence of hydrophilic silica nanoparticles. Colloids Surf. B Biointerfaces.

[50] Wang J., Zhu H. (2021). Interaction between polyene antifungal drug and saturated phospholipid monolayer regulated by calcium ions at the air-water interface. Colloids Surf. B Biointerfaces.

[51] Davies J.T., Rideal E.K. (1963). Interfacial Phenomena.

[52] Panda A., Possmayer F., Petersen N., Nag K., Moulik S. (2005). Physico-chemical studies on mixed oppositely charged surfactants: Their uses in the preparation of surfactant ion selective membrane and monolayer behavior at the air water interface. Colloids Surf. A Physicochem. Eng. Asp..

[53] Gopal A., Lee K.Y.C. (2006). Headgroup Percolation and Collapse of Condensed Langmuir Monolayers. J. Phys. Chem. B.

[54] Gzyl-Malcher B., Handzlik J., Klekowska E. (2012). Interaction of prazosin with model membranes—A Langmuir monolayer study. Bioelectrochemistry.

[55] Devterova J.M., Sokolov M.E., Buz’Ko V.Y., Repina I.N., Rudnov P.S., Panyushkin V.T. (2020). Subphase pH effect on the limiting molecular area of amphiphilic β-diketones in Langmuir monolayers. Mendeleev Commun..

[56] Wang J., Ma Y., Hou S. (2020). Effect of potassium ions at the different concentration on the interaction between AmB and the lipid monolayer containing cholesterol or ergosterol. Biochem. Biophys. Res. Commun..

[57] Botet-Carreras A., Montero M.T., Domènech Ò., Borrell J.H. (2019). Effect of cholesterol on monolayer structure of different acyl chained phospholipids. Colloids Surf. B Biointerfaces.

